# First report of brown widow spider sightings in Peninsular Malaysia and notes on its global distribution

**DOI:** 10.1186/s40409-015-0010-2

**Published:** 2015-05-09

**Authors:** Mustakiza Muslimin, John-James Wilson, Amir-Ridhwan M Ghazali, Kamil A Braima, John Jeffery, Fitri Wan-Nor, Mohamed E Alaa-Eldin, Siti-Waheeda Mohd-Zin, Wan S Wan-Yusoff, Yusoff Norma-Rashid, Yee L Lau, Mahmud Rohela, Noraishah M Abdul-Aziz

**Affiliations:** Department of Parasitology, Faculty of Medicine, University of Malaya, Kuala Lumpur, Malaysia; Institute of Biological Sciences, Faculty of Science, University of Malaya, Kuala Lumpur, Malaysia; Museum of Zoology, Institute of Biological Sciences, Faculty of Science, University of Malaya, Kuala Lumpur, Malaysia; Faculty of Veterinary Medicine, University Putra Malaysia, Serdang, Selangor Malaysia; Department of Civil Engineering, Faculty of Engineering, University of Malaya, Kuala Lumpur, Malaysia

**Keywords:** *Latrodectus geometricus*, Brown widow spider, Colonization, DNA barcoding, Envenomation, Global invasion, Invasive species, Medically important arthropods, Synanthropy

## Abstract

**Background:**

The brown widow spider (*Latrodectus geometricus* Koch, 1841) has colonised many parts of the world from its continent of origin, Africa. By at least 1841, the species had successfully established populations in South America and has more recently expanded its range to the southern states of North America. This highly adaptable spider has been far more successful in finding its niche around the world than its famous cousins, the black widow, *Latrodectus mactans,* found in the south-eastern states of North America, and the red-back, *Latrodectus hasselti*, found mostly in Australia, New Zealand and Japan.

**Methods:**

We performed an extensive web search of brown widow sightings and mapped the location of each sighting using ArcGIS. Specimens reputedly of the species *L. geometricus* were collected at three localities in Peninsular Malaysia. The spiders were identified and documented based on an examination of morphological characteristics and DNA barcoding.

**Results:**

The spiders found in Peninsular Malaysia were confirmed to be *Latrodectus geometricus* based on their morphological characteristics and DNA barcodes. We recorded 354 sightings of the brown widow in 58 countries, including Peninsular Malaysia.

**Conclusion:**

Reports from the Americas and the Far East suggest a global-wide invasion of the brown widow spider. Herein we report the arrival of the brown widow spider in Peninsular Malaysia and provide notes on the identification of the species and its recently expanded range.

## Background

The widow spiders comprise 30 species in the genus *Latrodectus* Walckenaer, 1805 [1]. They earned the name “widow” because the female eats the male after mating. However, this behavior has been only conclusively documented for one species, the red-back spider (*L. hasselti* Thorell, 1870) [2]. Black widow [*L. mactans*, (Fabricius 1775)] envenomation can cause death in humans; however, lethality is less than 1% [3,4]. Human mortality caused by the red-back spider, native to Australia and New Zealand, has never been reported, perhaps in part because these two countries have an extensive supply of antivenom [5,6]. Human death resulting from envenomation by the lesser-known brown widow (*L. geometricus* Koch, 1841) was reported in Madagascar in 1991. However, the identity of the spider, consequences of delayed medical intervention and the exact details of the case, whose report is in French, remain in question [7]. Furthermore, medical conditions associated with spider bites are often over-diagnosed and misdiagnosed [8]. The brown widow is known by many arachnologists to be nonaggressive and usually bites only when threatened. Like most widow spiders, it avoids people and prefers the shelter of its protective retreat. If the retreat is disturbed, the spider often jumps from its web to the ground, retracts its legs and plays dead, in a behavior known as thanatosis [9].

Reports from the Americas to the Far East suggest a recent global-wide invasion of the brown widow spider and numerous sightings of brown widows have been reported in Central Asia and the Middle East [10-16]. The Department of Parasitology at the University of Malaya Faculty of Medicine, Kuala Lumpur, is monitoring this invasion and is contacted periodically by concerned local citizens reporting unusual spiders in their homes. These concerns are likely unwarranted since only one single case of a venomous spider (*Lampropelma violaceopedes* Abraham, 1924) biting a human has been reported in Southeast Asia [17]. However, rapid urbanization in East Asia and the region’s bustling economic trade and growing population may promote colonization by synanthropic widow spiders as specimens “hitchhike” in containers to densely populated areas [1,18,19]. This paper reports the arrival of the brown widow spider in Peninsular Malaysia and provides notes on the identification of the species and its global distribution.

## Methods

### Global distribution of *Latrodectus geometricus*

Global records of *L. geometricus* were compiled from the scientific literature and popular media through web searches, together with GPS coordinates obtained directly from the records or inferred as precisely as possible from the stated locations. These records were then mapped using ArcGIS 9.2 [20].

### *Latrodectus geometricus* in Peninsular Malaysia

Acting on calls from the general public, spider specimens reputedly of the species *L. geometricus* were collected at three localities in Peninsular Malaysia: Penang (5°24′00″N, 100°14′20″E), at a private residence after obtaining permission from the owners; on the roadside, near food stalls in a residential area in Selangor (3°20′N, 101°30′E); and in a private vehicle in Johor (1°29′14″N, 103°46′52″E) after obtaining the permission of the vehicle owner. Permission to collect spiders in Peninsular Malaysia was approved by the Department of Wildlife and National Parks of Peninsular Malaysia, commonly known as PERHILITAN (application number: JPHL&TN(IP): 80-4/2 Jld16).

Spiders were provisionally identified by examining the palps, epigynum, geometrical markings on the underside of the abdomen and spherical spikey off-white egg sacs [21]. As a member of the family Theridiidae, *L. geometricus* has four pairs of eyes positioned in two parallel rows, a comb feet arranged in a comb-like row of bristles on the tarsi of the hind legs, and distinctive paired spermathecae with coiled copulatory ducts [21-24]. Similarly to some other widow spiders, brown widows have a characteristic hourglass-shaped streak on the underside of the abdomen which varies from a pale to dark orange as the spider matures. Females are significantly larger than males (leg length 30-40 mm compared with 16-20 mm in males).

DNA was extracted from whole spiders using a NucleoSpin tissue kit (Macherey-Nagel) following the procedures recommended by the manufacturer. We PCR-amplified the “DNA barcode” fragment of the mitochondrial cytochrome c oxidase subunit I (*COI*) gene (mtDNA) using the primer combination LepF1/LepR1 and standard thermocycling conditions [25]. The PCR product was sequenced in both directions using the PCR primers by a local company (MyTACG Bioscience, Kuala Lumpur). The resulting sequences were edited and combined with all COI sequences from *Latrodectus* available on the GenBank and analyzed using the neighbor-joining method and MEGA 6 software [26]. The DNA sequences and associated information about the specimens (photographs, collection date and locality) can be found in the public dataset DS-LATRO on the Barcode of Life Datasystems’ (BOLD) website (http://www.boldsystems.org), and also on GenBank (http://www.ncbi.nlm.nih.gov/genbank; accession numbers: KF227386-KF227396).

## Results

**Figure 1:**
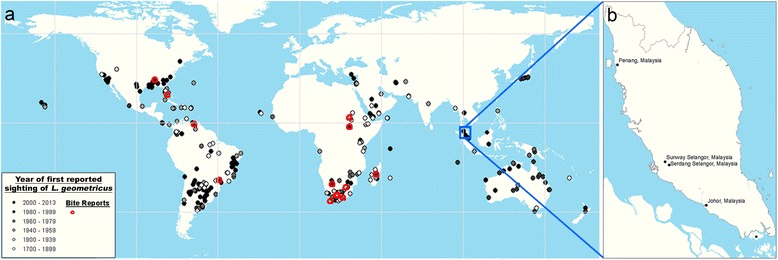
Distribution of reported sightings of *Latrodectus geometricus.* **(a)** Map showing the global distribution of *Latrodectus geometricus* [1,2,7,11,12,19,21-24,27-127]. **(b)** Locations in the northwest, central and south of Peninsular Malaysia with new records of the brown widow spider.

**Figure 2:**
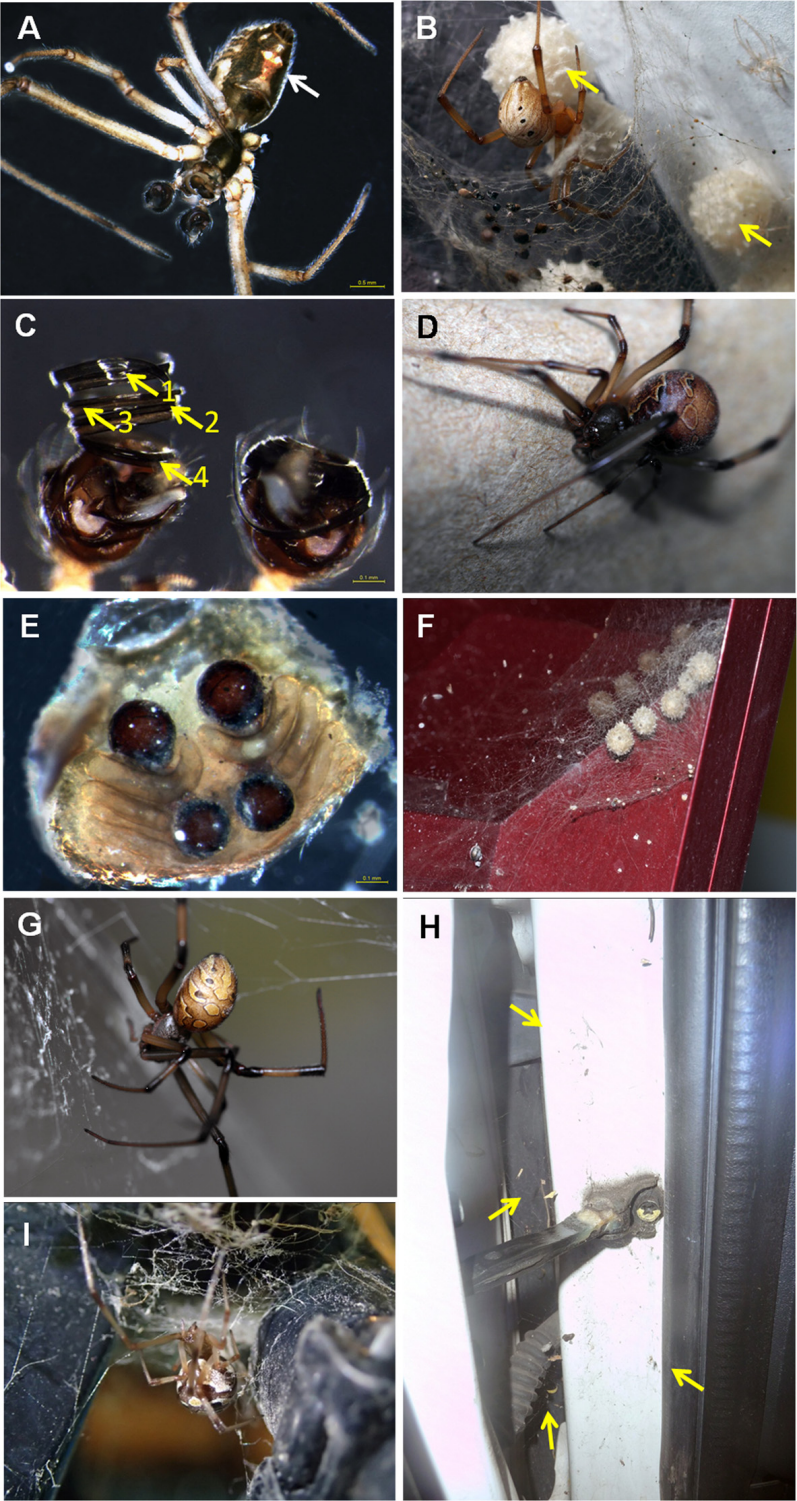
Common morphological characteristics of *Latrodectus geometricus* according to Koch (1841). **(A)** The hourglass-shaped streak on the underside of the abdomen (male, Johor, Malaysia). **(B)** Dome-shaped abdomen typical of a juvenile Theridiidae and the spiky spherical egg sacs (Penang, Malaysia). **(C)** Embolus inside the palp of the male spider showing four coils. **(D)** A brown widow caught in central Peninsular Malaysia bearing darker and more spherical features on its abdomen. **(E)** The epigynum, characteristic of females, with two pairs of spermathecae located on the underside of the abdomen. **(F)** Spiky spherical egg sacs lined in a row on a window sill of a house (Penang, Malaysia). **(G)** The dome-shaped abdomen of a female brown widow from the northwest of Peninsular Malaysia bearing lighter features on its abdomen. **(H)** Egg sacs, moulted skin and live and dead juvenile/adult *L. geometricus* (yellow arrows) being surrounded by its cobweb. **(I)** Dorsal aspect of abdomen with distinct pattern surrounded by cobweb, found in and around bicycle compartment.

**Figure 3:**
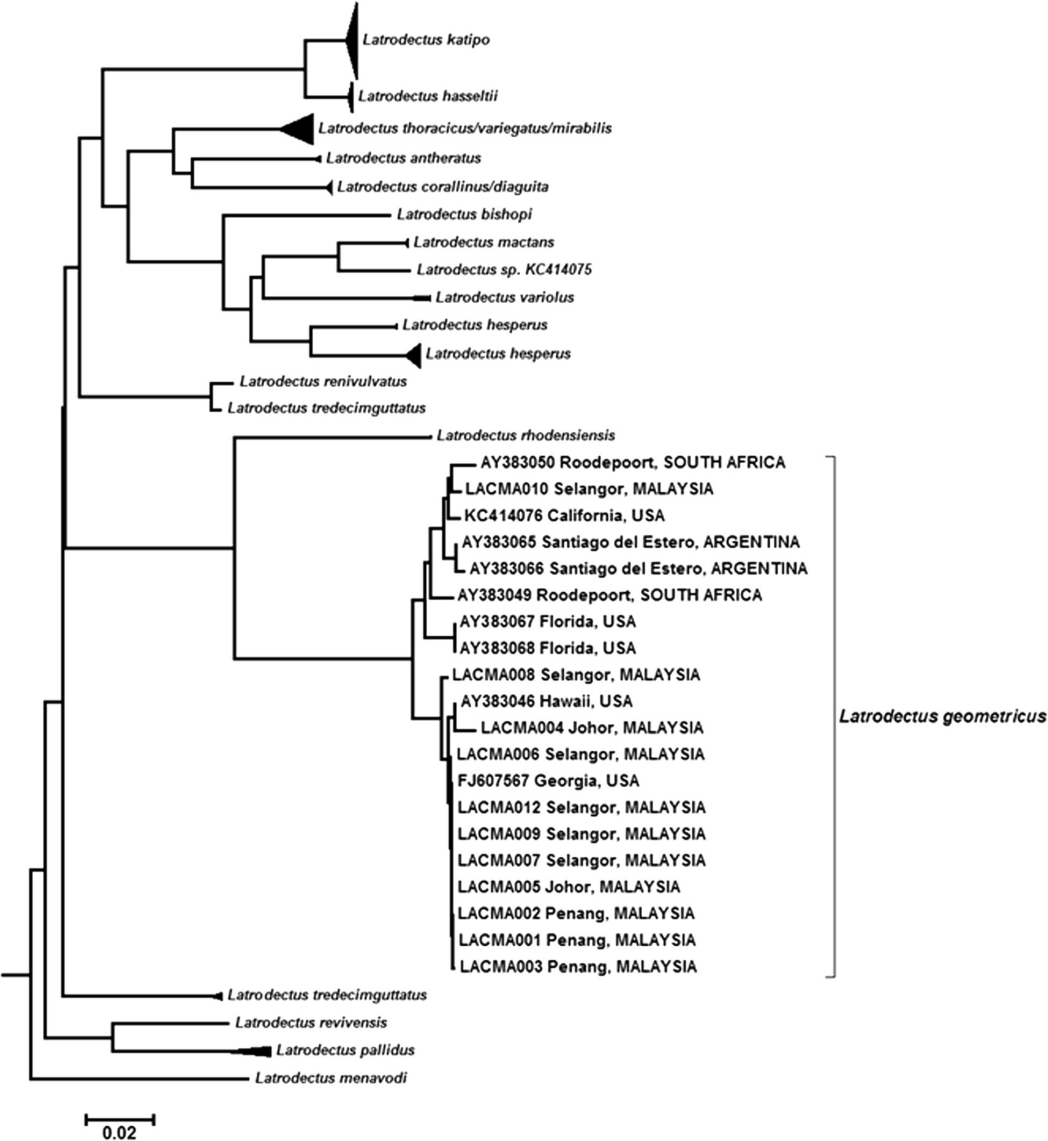
Neighbor-joining tree showing K2P distances between newly sequenced DNA barcodes from spiders collected in Peninsular Malaysia (codes: LACMA00XX) and publicly available sequence data for *Latrodectus geometricus* collected worldwide (GenBank accession numbers by locality).

We compiled 354 records of *L. geometricus* sightings from 117 sources (Figure 1) and plotted the locations onto a world map (Figure 1). The spiders collected in Penang, Selangor and Johor were confirmed as *L. geometricus* based on an examination of morphological characteristics (Figure 2) and DNA barcoding (Figure 3) and therefore added to the world map.

## Discussion

Although the description of this species is based on a specimen collected in Colombia, South America in 1841, *Latrodectus geometricus* is thought to be native to southern Africa and to have gradually expanded its range since the 1800s to cover both tropical and temperate regions of the world (Figure 1) [29]. Sightings of brown widows had already been reported in South America, North America, and the Middle East (Yemen in 1890) before 1900 [28,32,88]. Since then, sightings were reported in Saudi Arabia in 1959, Israel in 1983, Central Asia (Afghanistan in 2008 and Turkey in 2008), Southeast Asia (Indonesia in 1950, the Philippines in 1950 and Singapore in 2006), Japan in 1995, and Australia in 1987 [15,51,52,73,75,80,86,95,107]. Although the presence of the brown widow has yet to be reported in Europe, with the exception of Turkey, the species is extending its range into temperate North America with recent reports from southern US states such as Texas, North Carolina and Mississippi [128].

Although the *L. geometricus* specimens found in Peninsular Malaysia were easily identifiable due to their morphological characteristics, the findings were confirmed using DNA barcoding. The brown widows collected in the northwest (Penang) and south (Johor) of Peninsular Malaysia seemed to be morphologically similar to those reported in port cities in Japan, suggesting that Japan is the source of the infestation [127]. This would not be surprising, given the amount of trade between Japan and these two ports [129]. Penang is an international port, popularly known as the Pearl of the Orient, and therefore we suspect that *L. geometricus* was accidentally imported.

Upon arrival, *L. geometricus* is known to colonize urban areas especially in and around homes and gardens, which is consistent with our observations of brown widow webs and eggs in both well-lit and dark areas around windows, ceilings and car door hinges [1,48,78,130]. Since *Latrodectus geometricus* was reported in Singapore, the proximity of Singapore and Johor suggests that the brown widow collected in Johor may have come from the former, whose population is likely to have originated in Japan [107]. The *L. geometricus* colony from Selangor (central Peninsular Malaysia) appeared to have two different morphotypes. However, no genetic differentiation was found in their DNA barcodes. The first was similar to those found in Penang and Johor, while the second had a spherical abdomen and was more similar to the brown widow reported in India and Brazil [103]. Phylogeographic studies of different gene regions together with searches for *L. geometricus* in other localities may shed some light on these findings. Following recent reports from South and North America, Peninsular Malaysia is the latest region to be occupied by the global invasion of the brown widow.

## Conclusions

The brown widow spider remains a potential concern and should be monitored. Reports from the Americas and the Far East suggest a global-wide invasion of the brown widow spider due to its far-reaching adaptability. The arrival of the brown widow spider in Peninsular Malaysia including identification of the species using both taxonomic and molecular methods was reported. Furthermore, its recently expanded range and its phylogeographic distribution were discussed in view of its impact on humans.

### Ethics committee approval

Permission to collect samples was granted by the Department of Wildlife and National Parks, Peninsular Malaysia (PERHILITAN): application number JPHL&TN(IP): 80-4/2 Jld16(24). This consent prohibits the collection of endangered or protected species.
